# A Genetically Encoded Picolyl Azide for Improved Live Cell Copper Click Labeling

**DOI:** 10.3389/fchem.2021.768535

**Published:** 2021-11-11

**Authors:** Birthe Meineke, Johannes Heimgärtner, Alexander J. Craig, Michael Landreh, Lindon W. K. Moodie, Simon J. Elsässer

**Affiliations:** ^1^ Science for Life Laboratory, Department of Medical Biochemistry and Biophysics, Division of Genome Biology, Karolinska Institutet, Stockholm, Sweden; ^2^ Ming Wai Lau Centre for Reparative Medicine, Stockholm Node, Karolinska Institutet, Stockholm, Sweden; ^3^ Drug Design and Discovery, Department of Medicinal Chemistry, Biomedical Centre, Uppsala University, Uppsala, Sweden; ^4^ Department of Microbiology, Tumor and Cell Biology, Science for Life Laboratory, Karolinska Institutet, Stockholm, Sweden; ^5^ Uppsala Antibiotic Centre, Uppsala University, Uppsala, Sweden

**Keywords:** genetic code expansion, amber suppression, noncanonical amino acid, bioorthogonal chemistry, click chemistry, copper catalyzed azide–alkyne cycloaddition (CuAAC)

## Abstract

Bioorthogonal chemistry allows rapid and highly selective reactivity in biological environments. The copper-catalyzed azide–alkyne cycloaddition (CuAAC) is a classic bioorthogonal reaction routinely used to modify azides or alkynes that have been introduced into biomolecules. Amber suppression is an efficient method for incorporating such chemical handles into proteins on the ribosome, in which noncanonical amino acids (ncAAs) are site specifically introduced into the polypeptide in response to an amber (UAG) stop codon. A variety of ncAA structures containing azides or alkynes have been proven useful for performing CuAAC chemistry on proteins. To improve CuAAC efficiency, biologically incorporated alkyne groups can be reacted with azide substrates that contain copper-chelating groups. However, the direct incorporation of copper-chelating azides into proteins has not been explored. To remedy this, we prepared the ncAA paz-lysine (PazK), which contains a picolyl azide motif. We show that PazK is efficiently incorporated into proteins by amber suppression in mammalian cells. Furthermore, PazK-labeled proteins show improved reactivity with alkyne reagents in CuAAC.

## Introduction

Genetic code expansion allows the expression of proteins with distinct chemical handles through the residue- or site-specific introduction of noncanonical amino acids (ncAAs). First established in *Escherichia coli*, genetic code expansion has been adapted to all domains of life (Chin, 2014; Chin, 2017; Brown et al., 2018). When incorporated into proteins, ncAAs can confer a plethora of different functionalities: posttranslational modifications, crosslinking, spectroscopic probes, and also bioorthogonal chemical handles for selective reactions in the cellular context (Sletten and Bertozzi, 2009; Lang and Chin, 2014; Drienovská and Roelfes, 2020). Bioorthogonal chemistries enable endless possibilities for further derivatizing ncAA-containing proteins in or on live cells with fluorophores, lipids, or affinity handles (Lang et al., 2012; Elliott et al., 2014; Lang and Chin, 2014; Peng and Hang, 2016; Li et al., 2020; Meineke et al., 2020). The copper-catalyzed azide–alkyne cycloaddition (CuAAC, also referred to as “click” chemistry), is a Cu(I)-dependent, fast, biorthogonal, and widely utilized reaction to form covalent bonds between alkyne and azide moieties (Rostovtsev et al., 2002; Tornøe et al., 2002; Hein and Fokin, 2010; Haldón et al., 2015; Li and Zhang, 2016). To circumvent the need for Cu(I) catalysis, strained alkynes have also been realized in so-called strain-promoted azide–alkyne cycloaddition (SPAAC) reactions (Beatty et al., 2010; Jewett et al., 2010). Alkyne and azide ncAAs, e.g., the methionine analogs azido-alanine, 6-azido-norleucine, and homopropargylglycine, can be used for CuAAC-mediated metabolic labeling. These ncAAs are substrates for the endogenous translation machinery, charged onto tRNA^Met^ by methionyl-tRNA-synthetase (or an engineered mutant) and stochastically incorporated into nascent proteins in response to the AUG codon (Saleh et al., 2019). Site-directed incorporation of ncAAs into proteins, on the other hand, requires reprogramming of one codon and introduction of a dedicated, engineered pair of tRNA and aminoacyl-tRNA-synthetase (aaRS) that is orthogonal to, i.e., not interfering with, the translation machinery of the host. A widely used strategy to reprogram a codon is amber suppression, as the amber codon (UAG) is the least abundant of the three stop codons in *E. coli* and mammalian cells. Two different tRNA/aaRS systems have been used to site specifically install azide moieties in eukaryotic cells: AzFRS has been engineered from *E. coli* TyrRS to accept azido-phenylalanine (AzF) (Chin et al., 2002; Liu et al., 2007; Ye et al., 2009; Ye et al., 2010). AzFRS is combined with an amber suppressor mutant of *Bacillus stearothermophilus* TyrT (*Bst* TyrT^CUA^) for AzF incorporation in the mammalian system, which has shown higher expression than the cognate *Eco*TyrT (Sakamoto et al., 2002; Liu et al., 2007). AzF is routinely used for UV-crosslinking studies [reviewed in (Coin, 2018)]; the azide is also reactive in CuAAC or other click reactions (Bundy and Swartz, 2010; Tian et al., 2014). An alternative tRNA/aaRS pair for amber suppression is the versatile pyrrolysine-tRNA (PylT) and pyrrolysine-tRNA-synthetase (PylRS) pair derived from methanogenic archaea, which is orthogonal across bacterial and eukaryotic hosts. *Methanosarcina mazei* PylT/RS (*Mma* PylT/RS)-mediated ncAA incorporation is efficient in mammalian cells, and a large number of active site mutants for incorporation of structurally diverse ncAAs have been described. The lysine-based ncAAs *N*-propargyl-L-lysine (ProK) and *N*-ε-([2-Azidoethoxy]carbonyl)-L-lysine (AzeoK) are efficiently incorporated with the *Mma* PylT/RS pair (Nguyen et al., 2009; Meineke et al., 2020). Thus, genetic incorporation of azides and alkynes has provided facile means to derivatize proteins using bioorthogonal CuAAC chemistry. However, the dependence on Cu(I) for catalysis has provided challenges in performing CuAAC in a cellular environment. Due to the sensitivity of Cu(I) ions toward oxidation in the presence of atmospheric oxygen, Cu(I) is typically generated *in situ* using stoichiometric amounts of sodium ascorbate as a reducing agent. Water-soluble Cu(I) ligands, such as tris(hydroxypropyltriazolylmethyl)amine (THPTA), have greatly improved biocompatibility of CuAAC by effectively complexing Cu(I), enhancing reaction speed at low Cu(I) concentrations, while inhibiting both the reoxidation of Cu(I) to Cu(II) and the production of reactive oxygen species (Hong et al., 2009, 2010). A complementary approach to increase biocompatibility of CuAAC is the use of “copper-chelating azides,” such as picolyl azide (Uttamapinant et al., 2012; Kuang et al., 2010; Brotherton et al., 2009). Uttamapinant and others have demonstrated that positioning the azidomethyl group adjacent to the pyridine nitrogen significantly increases its reactivity in the presence of low Cu(I) concentrations, presumably by increasing the local concentration of the catalyst (Uttamapinant et al., 2012). Interestingly, copper-chelating azides improved reaction rates at low Cu(I) concentration synergistically with THPTA; hence, the combination of soluble ligands with picolyl azide allowed CuAAC to be performed on live cells at as low as 40 µM Cu(I) concentration, for which no toxicity was observed (Uttamapinant et al., 2012).

Despite the favorable properties of picolyl azide, genetic incorporation of copper-chelating azide moieties has not been reported in literature. Here, we synthesize a picolyl azide-lysine (PazK) ncAA that is readily incorporated using existing PylT/RS variants. We find that PazK has improved reactivity over simple azides in lysate and on live cells, especially at low Cu(I) concentrations, upgrading the repertoire of genetically encodable CuAAC reagents.

## Materials and methods

### Chemical synthesis of picolyl azide-lysine

Experimental procedures for the synthesis of PazK can be found in the supporting information.

### Commercial Non-canonical Amino Acids

4-Azido-L-phenylalanine (AzF, CAS: 33173-53-4, Santa Cruz Biotechnology) and (S)-2-amino-6-[(2-azidoethoxy)carbonylamino]hexanoic acid (AzeoK, CAS: 1994331-17-7, Iris Biotech) were prepared as 100 mM stock solutions in 200 mM NaOH and 15% DMSO (w/v), and used at the final concentrations indicated.

### DNA constructs

The constructs for expression of *Mma* PylT/RS wild type (RRID: Addgene_140009) and AF (RRID: Addgene_140023) variants as well as the sfGFP150TAG reporter constructs (RRID: Addgene_154766) were described previously (Meineke et al., 2018, 2020). We generated analogous constructs for AzFRS with four repeats of *Bst* TyrT^CUA^ (RRID: Addgene_140018 and Addgene_174891). The plasmids share a common architecture and are here collectively referred to as “pAS” (Amber Suppression) plasmids: the aaRS, reporter or gene of interest are controlled by an EF1 promoter and followed by an IRES that allows expression of a downstream selection marker. A cassette with four tandem repeats of the tRNA gene, controlled by 7SK Pol III promoter, is placed upstream of the EF1 promoter in antisense orientation. All DNA constructs were verified by Sanger sequencing.

### Cell culture and transfection

HEK293T cells were maintained in Dulbecco’s modified Eagle’s medium (DMEM, GlutaMAX^TM^, Thermo) supplemented with 10% (v/v) FBS at 37°C and 5% CO_2_ atmosphere. For transient transfection, 1.5–2.0 × 10^5^ cells/ml were seeded 24 h before transfection with TransIT-LT1 (Mirus) according to the instructions of the manufacturer. ncAAs were added at the time of transfection, and cells were harvested after 24 h.

### Intact mass spectrometry

A modified transfection protocol was used for larger-scale GFP expression for bead purification, increasing the amount of total DNA to 6 μg (*Mma* PylT/RS AF and PylT/sfGFP150TAG at 2 + 8 ratio) per ml culture and transfecting 5.0–8.0 × 10^5^ cells/ml with 2 μg of polyethylenimine (PEI) per μg of DNA. PazK was supplemented to 0.5 mM at transfection and until harvest after 6 days. Cells were lysed in RIPA buffer supplemented with 1× cOmplete protease inhibitor (Roche). The insoluble fraction was removed by centrifugation. Expressed GFP was captured on GFP-Trap_MA magnetic beads (ChromoTEK), washed with RIPA buffer and PBS, and eluted in 1% (v/v) acetic acid.

Purified GFP samples were desalted and rebuffered into 100 mM ammonium acetate, pH 7.5, using ZebaSpin columns with a 7-kDa cutoff (Thermo). Samples were directly infused into an Orbitrap Fusion Tribrid mass spectrometer equipped with an offline nanospray source using borosilicate capillaries (Thermo). The capillary voltage was 1.5 kV, and the pressure in the ion-routing multipole was maintained at 0.11 torr. Spectra were acquired in the Orbitrap mass analyzer operated in high mass mode at a resolution of 60.000 between 1,000 and 4,000 m*/z*. Data were analyzed using Excalibur (Thermo).

### Live cell imaging for GFP expression

GFP-expressing HEK293T cells were imaged in a ZOE Fluorescent Cell Imager (BioRad).

### Bioorthogonal labeling in lysate

HEK293T cells were transfected, cultured in the presence of 0.25 mM ncAA for 24 h and lysed in RIPA buffer with 1× cOmplete protease inhibitor (Roche). The insoluble fraction was removed by centrifugation. CuAAC was carried out on equal volume aliquots in 1 mM CuSO_4_, 1 mM TCEP, 100 µM THPTA, and 1 µM AF647 dye (AF647-Alkyne or AF647-Picolyl Azide (Jena Bioscience)) for 1 h at 24°C, 450 rpm followed by incubation at 4°C overnight. Samples were separated on 4%–20% Tris-glycine gels (BioRad) and exposed for in-gel fluorescence at 630 nm in a GE AI600 imager and further analyzed by Western blot.

### Bioorthogonal labeling of surface receptor proteins on live cells

Transfected HEK293T cells were grown in the presence of 0.25 mM PazK or 0.25 mM AzeoK for 24 h. Cells were washed with PBS and labeled with 5 µM AF647-alkyne dye (Jena Bioscience), 10–50 µM CuSO_4_, 50–250 µM THPTA in 2.5 mM ascorbic acid (from a freshly prepared 100 mM stock) for 10 min at room temperature (Hong et al., 2010). Cells were collected in cold PBS, spun down, and lysed in PBS 0.1% (v/v) triton X-100 supplemented with 1× cOmplete protease inhibitor (Roche). Aliquots were separated on 4%–20% Tris-glycine gels (BioRad) and exposed for in-gel fluorescence at 460 and 630 nm in a GE AI600 imager and further analyzed by Western blot.

### Labeling of surface receptor proteins on live cells for fluorescence microscopy

Transfected HEK293T were grown on poly-L-lysine-coated 18-well imaging slides (Ibidi) in the presence of 0.25 mM PazK or AzeoK for 24 h. Cells were washed with PBS and labeled with 5 µM alkyne dye (AFdye 647 alkyne, Jena Bioscience) in 50 µM CuSO_4_, 250 µM THPTA, and 2.5 mM ascorbic acid for 10 min at room temperature. Subsequently, the cells were washed with PBS, counterstained with 2 µM Hoechst33342 (Life Technologies) in PBS for 30 min, washed again, and fixed in 4% formaldehyde for 10 min. The cells were washed and imaged in PBS on a Nikon Eclipse Ti2 inverted widefield microscope, using a ×20 (0.75 NA) objective and filter sets for DAPI and Cy5 fluorescence.

### SDS-PAGE and Western blot

Aliquots of cell lysates were separated on 4–20% Tris-glycine gels (BioRad) and transferred to nitrocellulose membranes. Expression of GFP reporter and FLAG-aaRS was confirmed by immunoblotting with antibodies against GFP (Santa Cruz, RRID:AB_627695), HA-HRP (Roche, RRID:AB_390917), FLAG-HRP (Sigma, RRID:AB_439702), GAPDH (Millipore, RRID:AB_10615768), and corresponding secondary HRP-conjugated antibodies when needed (BioRad, RRID:AB_11125936 and Invitrogen, RRID:AB_2534727). Quantitative analysis of gel lanes was performed using ImageJ software.

## Results

### Synthesis of picolyl azide-lysine

**FIGURE 1:**
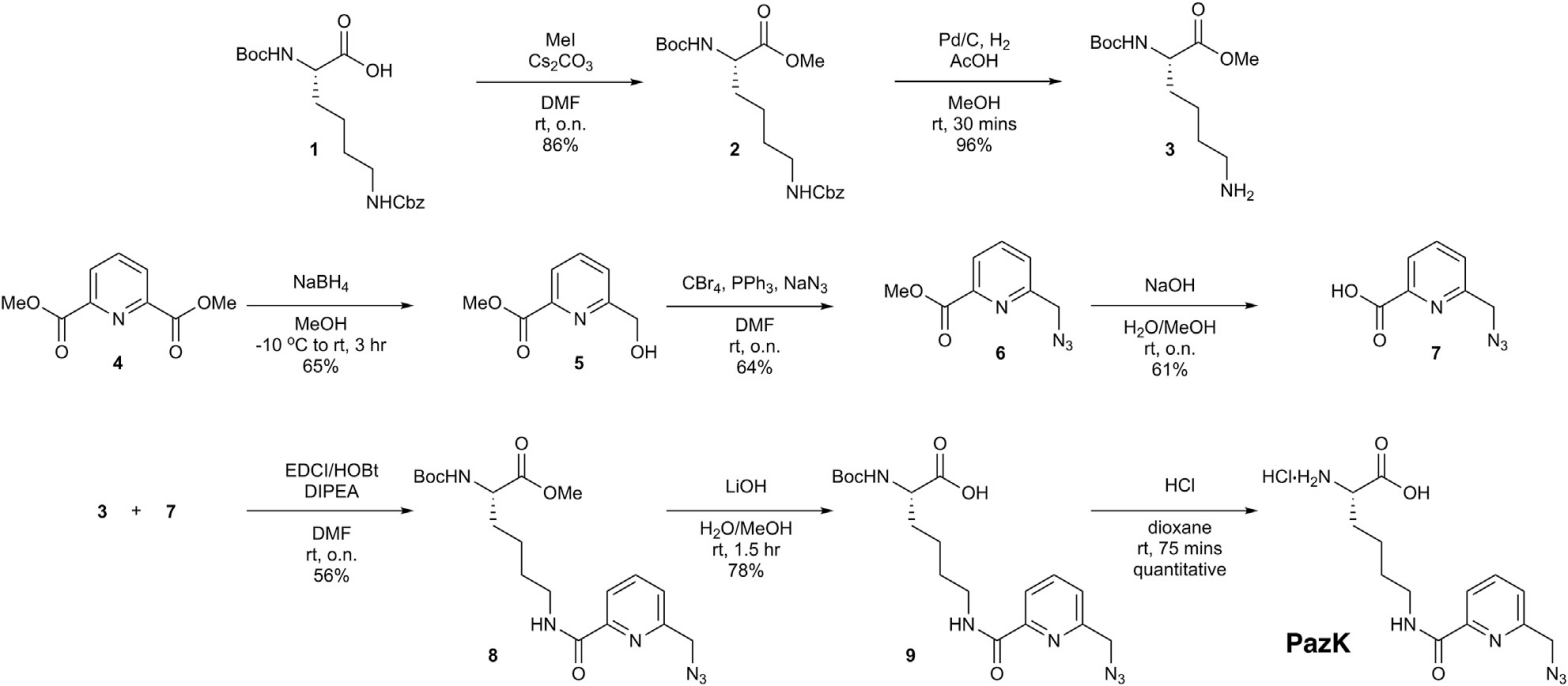
Chemical synthesis of picolyl azide-lysine (PazK).

To synthesize picolyl azide-lysine (PazK), two building blocks were required, lysine derivative **3** and azide **7** (Figure 1). The synthesis of **3** commenced with orthogonally protected *N*α-Boc-*N*ε-Cbz-Lysine **1**. After methylation under standard conditions, hydrogenation afforded **3** (Schnell et al., 2020), which contains the free side chain amine, in 83% yield over two steps. To access azide **7**, dipicolinic acid dimethyl ester **4** was selectively reduced with NaBH_4_ to alcohol **5** in 65% yield. Installation of the requisite azide functionality was effected using a one-pot process where the hydroxyl group of **5** was converted into the corresponding alkyl bromide (PPh_3_ and CBr_4_), followed by displacement with sodium azide without isolation of the bromide intermediate. Subsequent ester hydrolysis under basic conditions afforded carboxylic acid **7** (Hanna et al., 2017). With building blocks **3** and **7** in hand, amide formation was performed using standard conditions (EDCI, HOBt, DIPEA in DMF). Lithium hydroxide-mediated ester hydrolysis, followed by Boc deprotection under acidic conditions afforded the desired PazK, as the hydrochloride salt.

### *Methanosarcina mazei* pyrrolysine-tRNA-synthetase AF active site mutant allows incorporation of picolyl azide-lysine into proteins

**FIGURE 2:**
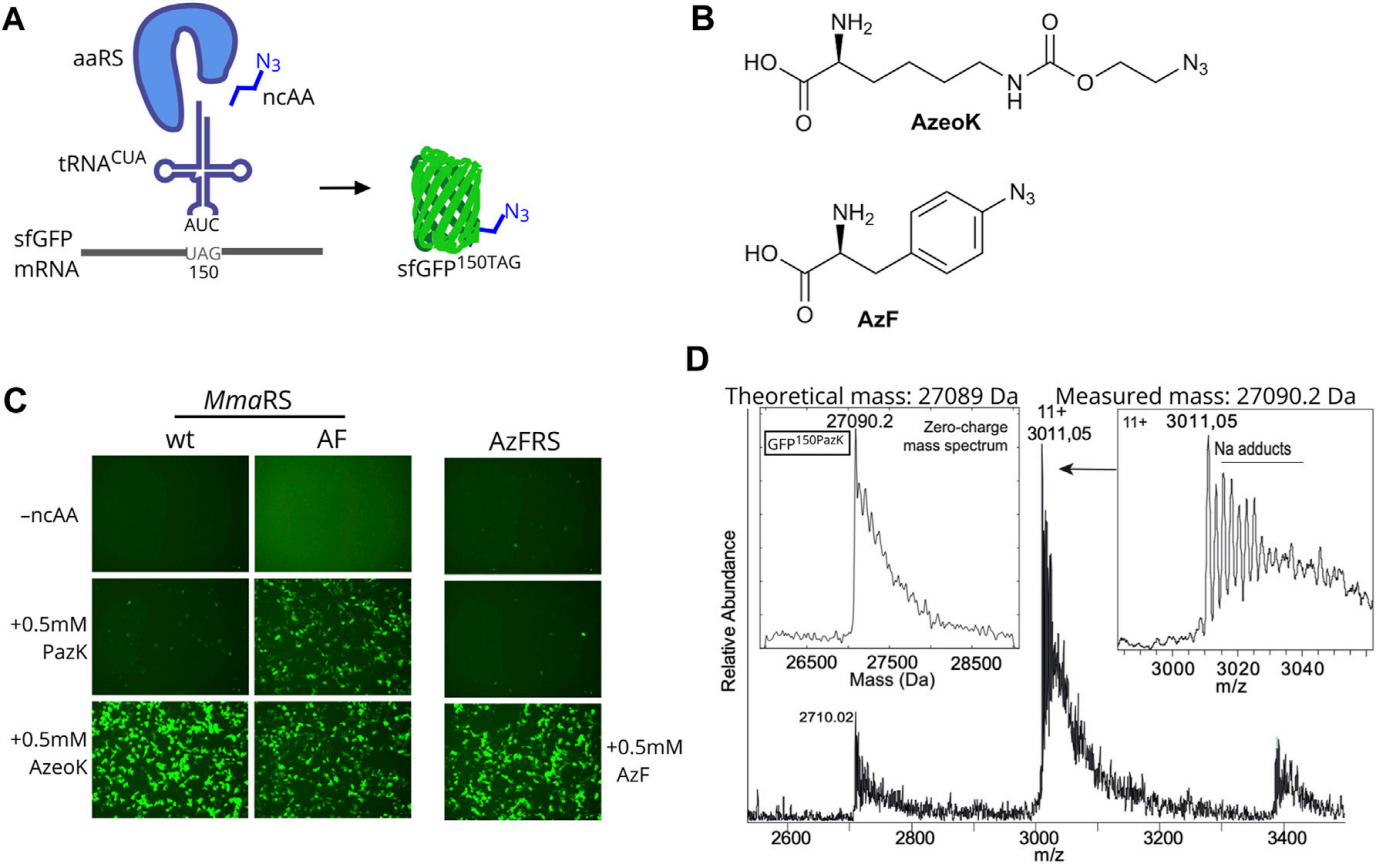
Amber suppression mediated PazK incorporation into protein in mammalian cells. **(A)** Schematic depiction of noncanonical amino acid (ncAA) incorporation into GFP. Expression of an orthogonal tRNA and aaRS pair allows incorporation of ncAAs with azide side chains into the GFP reporter in response to an amber codon (UAG in the mRNA). **(B)** Chemical structures of (S)-2-amino-6-[(2-azidoethoxy)carbonylamino]hexanoic acid (AzeoK) and 4-azido-L-phenylalanine (AzF). **(C)** Live-cell imaging of HEK293T cells transfected with *Methanosarcina mazei* pyrrolysine-tRNA/RS (*Mma* PylT/RS) wt, *Mma* PylT/RS AF, or *Bacillus stearothermophilus* (*Bst*) TyrT/AzFRS and cognate tRNA/GFP150TAG reporter plasmid (1 + 4 ratio) in the absence (–ncAA) or presence of 0.5 mM of the indicated ncAA. Images were taken 24 h posttransfection. **(D)** Intact mass determination of purified GFP containing 150PazK (incorporated with *Mma* PylRS AF in GFP150TAG).

Next, we needed to establish that PazK can be accepted as a substrate for tRNA aminoacylation by a tRNA^CUA^/aaRS pair orthogonal in mammalian cells. We used a GFP reporter with an amber codon at position 150, allowing the use of fluorescence as a readout for incorporation efficiency. If the ncAA added to the medium is accepted by the aaRS to aminoacylate the cognate tRNA^CUA^, the amber stop codon is suppressed, and full-length fluorescent GFP bearing PazK at position 150 (GFP^150PazK^) is produced (Figure 2A).

We tested the incorporation of PazK by *Mma* PylRS and its variant with mutations Y306A and Y384F: *Mma* PylRS AF (Yanagisawa et al., 2008). Wild-type *Mma* PylRS can accommodate a variety of ncAA substrates in its active site, but the Pyl binding pocket cannot accommodate large or bulky lysine adducts. The *Mma* PylRS AF mutant has been rationally designed to enlarge the ncAA binding pocket (Yanagisawa et al., 2008; Yanagisawa et al., 2019) and has enabled incorporation of lysine derivatives with aromatic and larger hydrocarbon rings (Borrmann et al., 2012; Nikić et al., 2014; Ge et al., 2016).

The *Mma* PylT/RS pairs were cotransfected with a PylT/sfGFP150TAG amber suppression reporter in HEK293T cells. We assayed AzeoK (Figure 2B) and PazK against a control with no ncAA, which showed no GFP fluorescence; AzeoK is an excellent substrate for wild-type *Mma* PylRS (Meineke et al., 2020) and, as expected, produced strong GFP fluorescence. PazK only yielded low GFP fluorescence with the same wild-type PylT/RS-transfected cells (Figure 2C). By adding AzeoK and PazK to *Mma* PylT/RS AF-expressing cells, we observed similar GFP fluorescence levels for both ncAAs (Figure 2C, right). For comparison, we also tested the incorporation of AzF (Figure 2B) and 6-azido-lysine (6AzK) (Supplementary Figure S1). AzF was efficiently incorporated in *Bst* TyrT^CUA^/AzFRS-expressing cells as judged by GFP fluorescence (Figure 2C), while 6AzK was not a substrate for *Mma* PylT/RS (Supplementary Figure S1). We further tested *Methanogenic archaeon ISO4-G1* (*G1*) PylT/RS and *G1* PylT/RS^Y125A^ pairs (Meineke et al., 2020) and found that wild-type *G1* PylRS accepted PazK with low efficiency, but *G1* PylRS^Y125A^ showed high incorporation efficiency for PazK (Supplementary Figure S2). Hence, we conclude that azide-bearing ncAAs can be incorporated well in mammalian cells with existing tRNA/aaRS pairs.

We further sought to confirm the selective incorporation and chemical stability of PazK in a target protein. Hence, we purified sfGFP^150PazK^ from HEK293T cells transfected with PylT/sfGFP150TAG and *Mma* PylT/RS AF and performed intact mass spectrometry. The calculated mass of 27,089 Da and determined mass of 27,090.2 Da were in agreement, confirming PazK incorporation and the stability of the picolyl-azide moiety in the cellular environment (Figure 2D).

### Copper-catalyzed azide–alkyne cycloaddition reactivity of GFP containing different azide-bearing non-canonical amino acids

**FIGURE 3:**
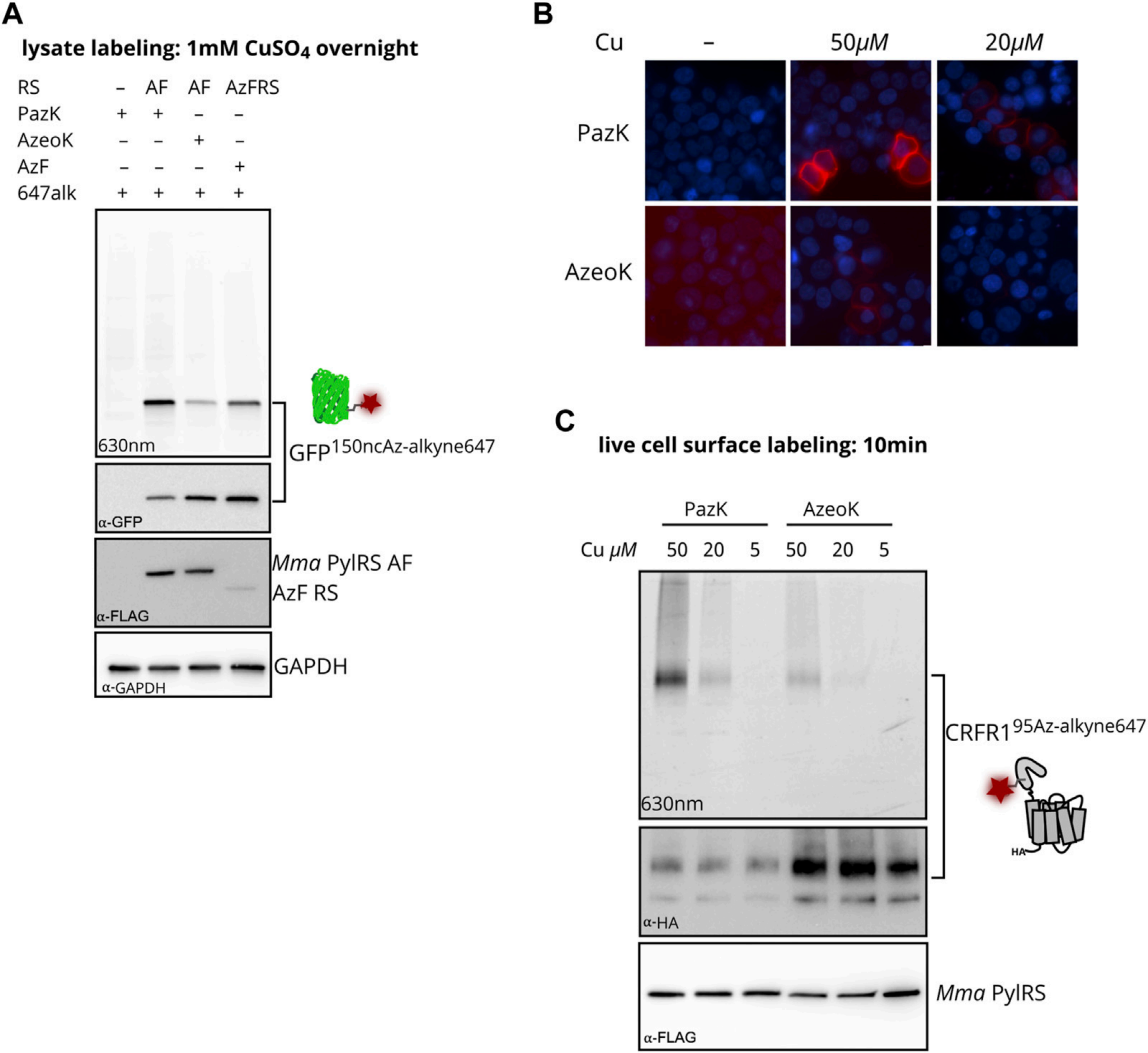
Incorporation of PazK allows copper-catalyzed azide–alkyne cycloaddition (CuAAC) at reduced copper concentrations. **(A)** CuAAC labeling of azide ncAAs in GFP in HEK293T cell lysate. Cells were transfected with *Mma* PylT/RS AF, or *Bst* TyrT/AzFRS and cognate tRNA/GFP150TAG reporter plasmid (1 + 4 ratio) and cultured in the absence (–ncAA) or presence of 0.25 mM of the indicated ncAA for 24 h. CuAAC labeling with 1 mM CuSO_4_, 1 mM TCEP, 100 µM THPTA, and 1 µM AF647-alkyne in cell lysate. Lysate aliquots were separated by SDS-PAGE and imaged for in-gel fluorescence. Immunostaining for GFP, FLAG-tagged aminoacyl-tRNA synthetase, and GAPDH loading control after membrane transfer of the same gel. **(B, C)** CuAAC labeling of CRFR1^95PazK^ and CRFR1^95AzeoK^ on the surface of live HEK293T cells. Cells were transfected with *Mma* PylT/RS AF and *Mma* PylT/CRFR1 95TAG reporter plasmid (1 + 4 ratio) and cultured in the absence (–ncAA) or presence of 0.25 mM of the indicated ncAA for 24 h. CuAAC labeling with 5–50 µM CuSO_4_, 25–250 µM THPTA, and 5 µM AF647-alkyne on live cells. **(B)** CuAAC-labeled cells were counterstained with Hoechst 33342, fixed in 4% formaldehyde for fluorescence microscopy. **(C)** Lysate aliquots were separated by SDS-PAGE and imaged for in-gel fluorescence. Immunostaining for HA-tagged CRFR1 and FLAG-tagged aminoacyl-tRNA synthetase after membrane transfer of the same gel.

To compare CuAAC labeling of the three azide-containing ncAAs, AzeoK, PazK, and AzF, we reacted GFP^150ncAA^ with fluorescent AF647-alkyne in HEK293T cell lysates after transient transfection of amber-suppressor tRNA/aaRS, using *Mma* PylT/RS AF for AzeoK and PazK and *Bst* TyrT/AzFRS for AzF (Figure 3A). In agreement with fluorescent imaging, anti-GFP Western blot confirmed the efficient incorporation of all three ncAAs, in the order AzF > AzeoK > PazK under the conditions used. For assessing the specificity of CuAAC reaction for the three ncAAs, we reacted a fluorescent dye, AF647-alkyne, via CuAAC in whole-cell lysate. Here, we chose traditional *in vitro* conditions with excess alkyne dye, high concentration of copper salt (1 mM), 100 µM THPTA, and long reaction time (1 h at RT followed by overnight incubation at 4°C) to reach a reaction end point. CuAAC AF647-alkyne yielded a single band corresponding to the size of GFP visible with in-gel fluorescence imaging at 630 nm (Figure 3A). No other bands are observed, confirming that all ncAA are orthogonal to (i.e., not incorporated by) the endogenous complement of aaRS enzymes. In principle, stoichiometric labeling should be observed under the given reaction conditions for the three azide-modified GFP proteins. However, despite the lower amount of total GFP produced, the signal for AF647-labeled GFP was strongest for PazK and weakest for AzeoK (corresponding to a roughly 7.5-fold higher AF647/GFP ratio for PazK compared to AzeoK) (Figure 3A). These results confirm that AzeoK and AzF are more efficiently incorporated, but suggest that the incorporated PazK has a higher CuAAC reactivity. There are several potential explanations for this observation: terminal azides can undergo reduction to amines, and aromatic azides are known to be photolabile; hence, some of the AzeoK and AzF azide moieties may have been eliminated in cellulo or upon lysis (Milles et al., 2012). On the other hand, natural Cu(I) chelating molecules in the crude lysate and reoxidation of Cu(I) to Cu(II) with atmospheric oxygen may deplete Cu(I) available for CuAAC under elongated reaction conditions. As an additional control, we performed an SPAAC reaction with dibenzocyclooctyne (DBCO)-TAMRA fluorescent dye in lysates of all the three azide-bearing GFP species and again observed an improved reactivity of PazK over AzeoK and AzF (Supplementary Figure S3). This further hinted at the decomposition of the AzeoK and AzF azide moieties in cellulo or upon cell lysis. In summary, these results, together with the intact mass (Figure 2D) suggest that PazK is favorably stable and reactive compared with other available azide ncAAs. Of note, SPAAC labeling with DBCO showed less specific labeling of the azide-bearing GFP and a number of background bands, in line with prior reports that SPAAC reactions are not strictly bioorthogonal due to side reactions with thiols (van Geel et al., 2012).

### Picolyl azide-lysine labeling with low copper concentrations

We moved on to investigate CuAAC labeling on live cells, where the concentration of added copper and labeling conditions must be optimized to find a compromise between reaction efficiency and adverse side effects to proteins and cells. CuSO_4_ concentrations of 50 µM in the presence of excess copper chelators have been successfully used for live cell CuAAC labeling, while higher concentrations have been shown to impact cell viability (Hong et al., 2010; Uttamapinant et al., 2012; Meineke et al., 2020). We incorporated PazK and AzeoK with *Mma* PylT/RS AF into an amber mutant of the class B GPCR corticotropin-releasing factor type 1 receptor (CRFR1 95TAG) (Coin et al., 2013; Serfling et al., 2018, Serfling et al., 2019). CuAAC with AF647-alkyne was performed with 5–50 µM CuSO_4_ and a fivefold excess of THPTA on the surface of live cells expressing CRFR1^95AzeoK^ or CRFR1^95PazK^ (Figures 3B, C). CRFR1^95AzeoK^ could be labeled with AF647-alkyne on the surface of live cells with 50 µM CuSO_4_, while AF647 fluorescence was barely detectable at 20 µM CuSO_4_ and undetectable at 5 µM CuSO_4_. CRFR1^95PazK^ yielded much stronger specific AF647 fluorescence at 50 and 20 µM CuSO_4_ despite the lower expression level. Incorporation efficiency of PazK and AzeoK into CRFR1 can be compared via detection of a C-terminal HA tag: at the same ncAA concentration, AzeoK addition allows much more efficient amber suppression (Figure 3C). Thus, PazK demonstrated greatly improved CuAAC reactivity over AzeoK at copper concentrations as low as 20 μM, while further reducing the copper concentration did not support CuAAC with either ncAA. Thus, we conclude that PazK, in combination with THPTA, allows efficient CuAAC reactions on live cells with minimal expected toxicity (Hong et al., 2010).

## Discussion

The discovery of strain-promoted inverse electron-demand Diels–Alder cycloaddition (SPIEDAC) has enabled versatile bioorthogonal reactions that are fast, efficient, and nontoxic in and on live cells (Lang and Chin, 2014; Nikić et al., 2014). As a result, CuAAC has become obsolete for many fluorescent labeling and chemical conjugation applications in cellular environments. However, CuAAC is exquisitely bioorthogonal as well as orthogonal to SPIEDAC and, thus, remains a universal choice for performing two orthogonal chemical conjugations in the same cellular environment (Nikić and Lemke, 2015). We have previously demonstrated orthogonal dual-color labeling of surface receptors on live cells combining SPIEDAC and CuAAC on genetic encoded *trans*-cyclooct-2-en-lysine (TCO*K) and ProK. Because PazK is a substrate for *Mma* PylRS AF and *G1* PylRS Y125A, it cannot be combined with TCO*K to form a second orthogonal ncAA pair for dual labeling. However, we note that ProK and PazK could be incorporated with the orthogonal *Mma* PylT/PylRS and *G1* hybT*/PylRS Y125A pairs (Meineke et al., 2020), hence, providing a route for installing site-specific alkynes and azides that could be employed for orthogonal fluorescent labeling as well as site-specific intramolecular or intermolecular crosslinking.

Currently, CuAAC reactions are limited to the cell surface because low intracellular Cu(I) concentration does not permit catalysis, and artificially raising copper concentrations within cells is likely toxic (Bevilacqua et al., 2014; Li et al., 2017). We determine a lower limit of 20 µM of copper for a successful CuAAC reaction with PazK on live cells. Synthesizing and screening additional structural variants of PazK may, in the future, improve incorporation efficiency and reactivity. For catalysis at even lower free copper concentrations, the copper-chelating properties of the azide ncAA could be enhanced by multivalent chelating ligands. For example, coordinating azides with two or three triazole rings have been shown, in principle, to enable intracellular CuAAC (Bevilacqua et al., 2014; Li et al., 2017). It will, thus, be an interesting challenge if PylT/RS variants can be identified that can accept larger copper-chelating azides and if availability of Cu(I) in the intracellular environment would be sufficient for catalysis.

## Data Availability

The original contributions presented in the study are included in the article/Supplementary Material. Further inquiries can be directed to the corresponding authors.
