# Supplementary material for: A Genetically Encoded Picolyl Azide for Improved Live Cell Copper Click Labeling
Source: Front Chem. 2021 Nov 11;9:768535. doi: 10.3389/fchem.2021.768535 (PMC8632528; doi:10.3389/fchem.2021.768535)
Supplement: Supplementary file 1 [file DataSheet1.PDF]

Supplementary Material

1 Supplementary Material and Methods

1.1 General chemistry methods

Thin-layer chromatography was performed on aluminum plates precoated with silica gel 60 F_{254}, with size of 0.2 mm from Merck. Compound were visualized with a UV-light, and stained with *p*-anisaldehyde. Column chromatography was performed with silica gel 60. Analytical high performance liquid chromatography was performed on a Dionez UltiMate 3000 HPLC system with a Bruker amazon SL ion trap mass spectrometer with detection by UV (diode array detector, 214, 254, and 280 nm) and electrospray ionization mass spectrometry using a Penomenex Kinetex C18 column (50 x 3.0 mm, 2.6 µm particle size, 100 Å pore size) with gradients of H_2O/MeCN/0.05% HCOOH as mobile phase at a flow rate of 1.5 mL/min. High resolution mass-spectra were recorded on either a QE Orbitrap or on a LTQ Velos Pro, with samples being analysed by direct infusion with electrospray ionization in positive mode. Data were collected at 140,000 resolution (at *m/z* 400) and 50 transients were co-added. ^{1}H NMR spectra were recorded at 400 MHz on a Varian Mercury Plus spectrometer. All spectra were recorded from samples in either $CDCl_3$ or MeOD, at room temperature in 5 mm NMR tubes. Chemical shifts are reported relative to the residual solvent peak at δ 7.26 for $CDCl_3$ or δ 3.34 for MeOD. Resonances were assigned as follows: chemical shift (multiplicity, number of protons, coupling constant(s)). Multiplicity abbreviations are reported by the conventions: s (singlet), br s (broad singlet), d (doublet), dd (doublet of doublets), t (triplet), app t (apparent triplet), app q (apparent quartet), app qd (apparent quartet of doublets), app p (apparent pentet), m (multiplet). Proton decoupled ^{13}C NMR spectra were recorded at 100 MHz on a Varian Mercury Plus spectrometer under the same conditions as for the ^{1}H NMR spectra. Chemical shifts have reported relative to the residual solvent peak at δ 77.16 for $CDCl_3$ or δ 49.86 for MeOD. All solvents and reagents were used as received.

1.2 DNA constructs

The constructs for expression of *Methanogenic archaeon ISO4-G1* (*G1*) PylT/RS^{Y125A} (RRID: Addgene_154769 and Addgene_154767) follow the pAS design and have been described (Meineke et al., 2020).

1.3 Quantification of GFP Expression.

Transfected HEK293T cells were grown in the presence of the indicated ncAA for 24 h. Cells were lysed in RIPA buffer without SDS with 1×cOmplete protease inhibitor (Roche); the insoluble fraction was removed by centrifugation. GFP bottom fluorescence of an aliquot was measured in a Tecan Infinite M200 pro plate reader (excitation 485 nm, emission 518 nm). All transfections were carried out in triplicate. Fluorescence measurements were normalized to total protein content of each sample as determined by Pierce BCA assay kit (FisherScientific) on the same sample.

1.4 Additional commercial ncAAs

*N*ε-[[(2-methyl-2-cyclopropene-1-yl)methoxy]carbonyl-L-lysine (CpK, CAS:1610703-09-7, SiChem), axial trans-cyclooct-2-ene-L-lysine (TCO*K, CAS:1801936-26-4, SiChem), *N*-

propargyl-L-lysine (ProK, CAS:1428330-91-9, Iris Biotech), *N*ε-Azido-L-lysine (6AzK, CAS: 1454334-76-9, Iris Biotech) were prepared as 100 mM stock solutions in 200 mM NaOH and 15 % (w/v) and used at the final concentrations indicated.

1.5 SPAAC lysate labeling with DBCO-TAMRA

HEK293T cells were transfected, cultured in the presence of 0.25 mM ncAA for 24 hours and lysed in RIPA buffer with 1xcOmplete protease inhibitor (Roche). The insoluble fraction was removed by centrifugation. Strain-promoted azide-alkyne cycloaddition (SPAAC) reaction was carried out on equal volume aliquots with 1 uM dibenzylcyclooctyne-PEG4- 5/6-tetramethylrhodamine (DBCO-TAMRA, Jena Bioscience) for 30 min on ice. Samples were separated on 4-20 % Tris-glycine gels (BioRad) and exposed for in-gel fluorescence at 520 nm in a GE AI600 imager and further analysed by Western blot.

Meineke, B., Heimgärtner, J., Eirich, J., Landreh, M., and Elsässer, S. J. (2020). Site-Specific Incorporation of Two ncAAs for Two-Color Bioorthogonal Labeling and Crosslinking of Proteins on Live Mammalian Cells. *Cell Rep.* 31, 107811. doi:10.1016/j.celrep.2020.107811.

2 Supplementary Figures

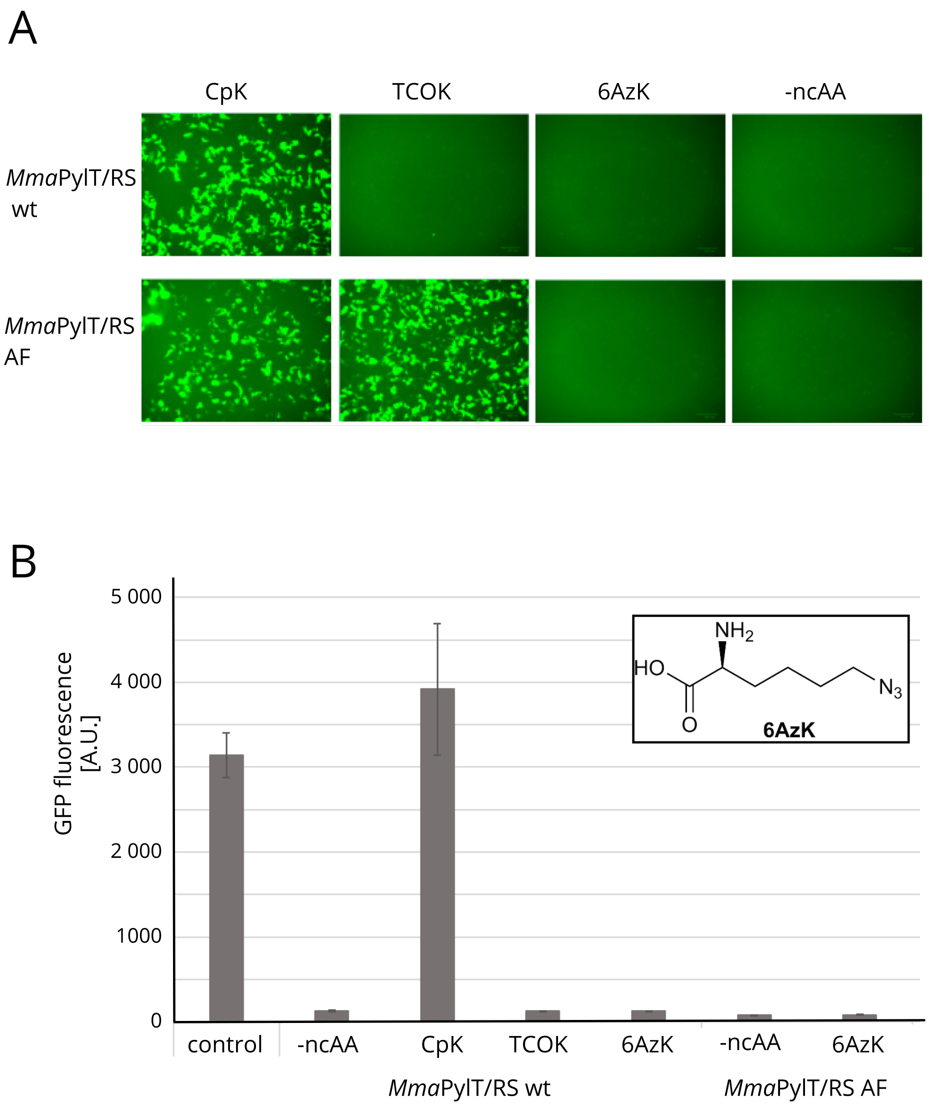

Supplementary Figure 1 - 6-azido-lysine (6AzK) is not a substrate for *Mma*PylT/RS

A) Live-cell imaging of HEK293T cells transfected with *Mma* PylT/RS wt or *Mma* PylT/RS and PylT/GFP150TAG reporter plasmid (1+4 ratio) in the absence (–ncAA) or presence of 0.2 mM CpK, 0.1 mM TCO*K or 0.5 mM 6AzK. Images were taken 24 h post transfection.

B) Fluorescence plate reader assay from HEK293T cell lysates transiently transfected in a 4:1 ratio with a GFP150TAG reporter *Mma* PylT/RS or *Mma* PylT/RS AF fluorescence is shown as in arbitrary units. As positive control a GFP construct without a TAG stop codon was included in the same experiment. Cells were grown for 24 h in the absence (–ncAA) or presence of one of the following ncAAs: 0.2 mM CpK, 0.1 mM TCO*K and 0.5 mM 6AzK. Data shown is the mean fluorescence calculated from triplicate transfections for each condition and error bars show standard deviation of the triplicate measurements.

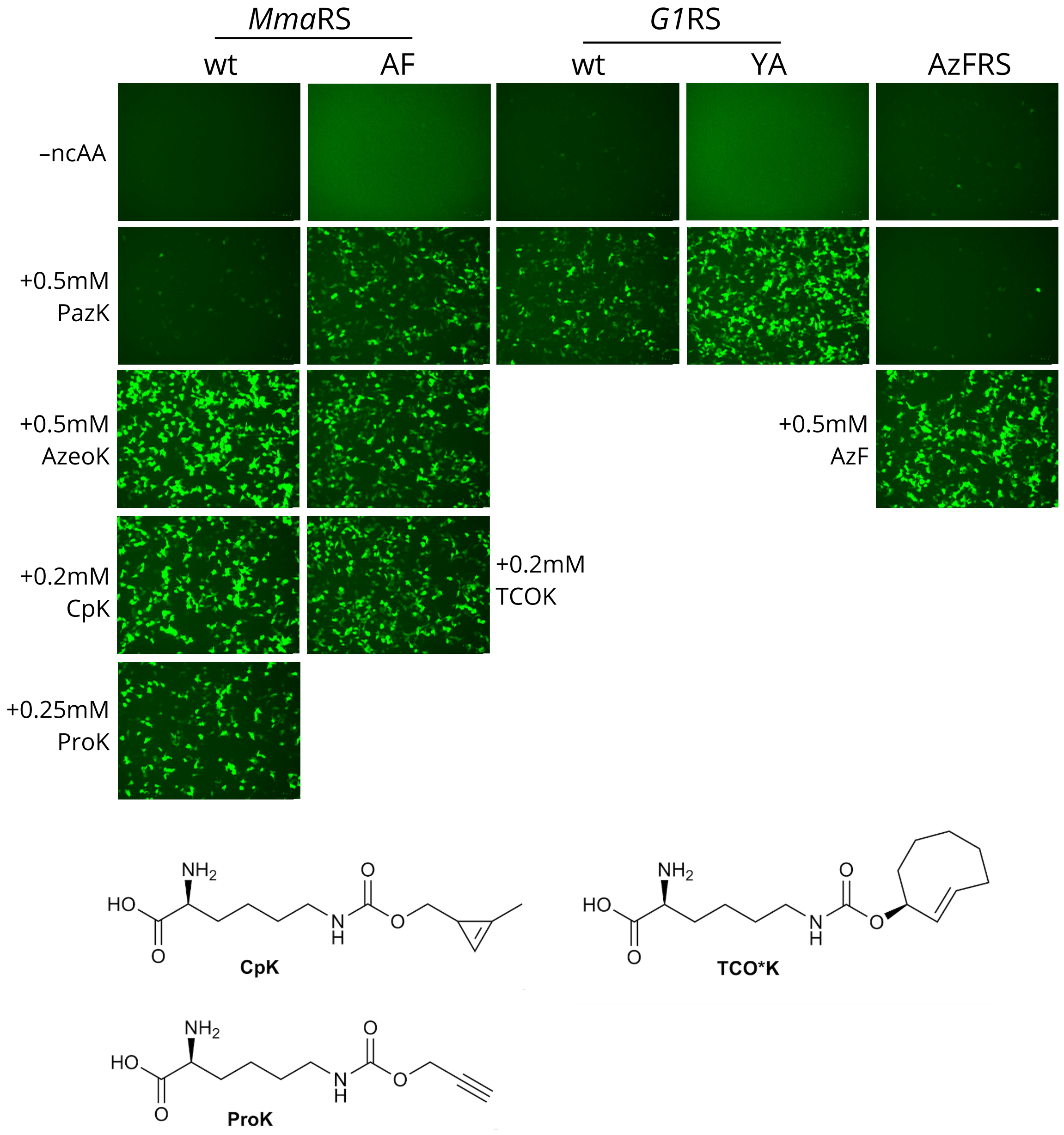

Supplementary Figure 2 - PazK is a good substrate for *G1*PylT/RS YA.
Expansion of Figure 2c. Live-cell imaging of HEK293T cells transfected with *Mma* PylT/RS wt, *Mma* PylT/RS AF, *G1*PylT/RS, *G1*PylT/RS YA or *Bst* TyrT/AzFRS and cognate tRNA/GFP150TAG reporter plasmid (1+4 ratio) in the absence (–ncAA) or presence of 0.5 mM of the indicated ncAA. Images were taken 24 h post transfection. Scale bars correspond to 100 µm. Chemical structures of the additional ncAAs CpK, TCO*K and ProK are shown.

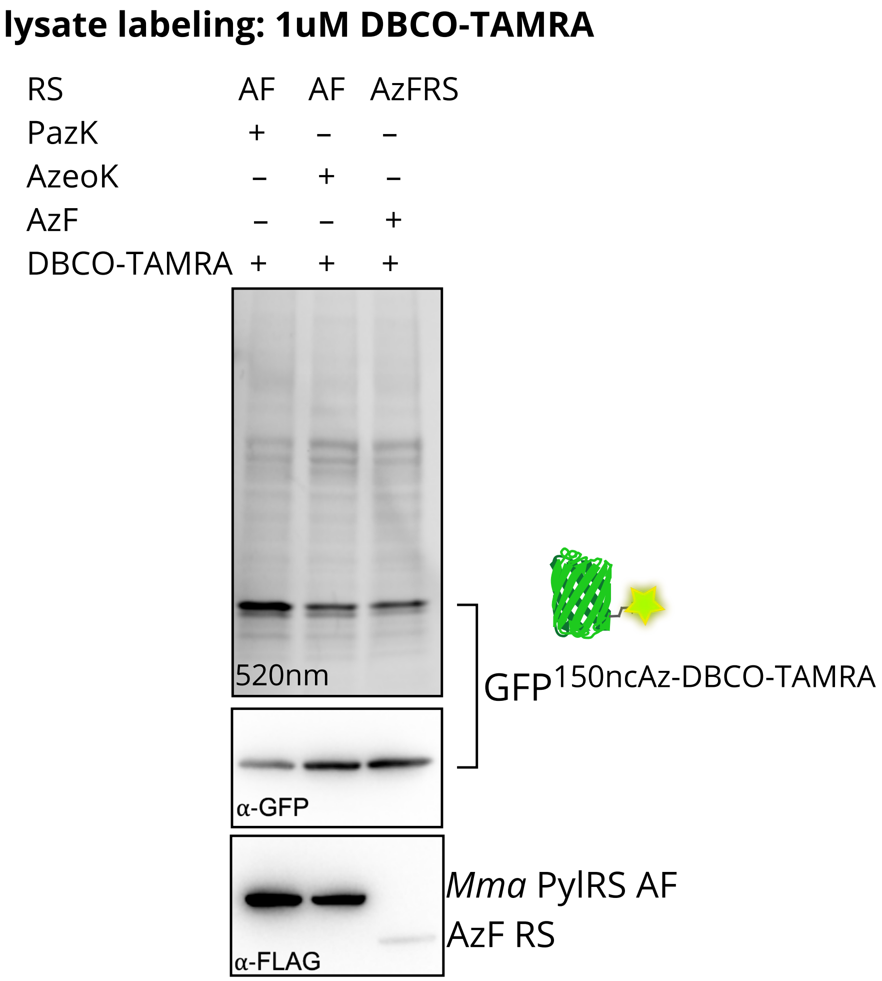

Supplementary Figure 3 - SPAAC lysate labeling of GFP^{150ncAA} with DBCO-TAMRA. SPAAC labeling of azide ncAAs in GFP in HEK293T cell lysate. Cells were transfected with *Mma* PylT/RS wt, *Mma* PylT/RS AF or *Bst* TyrT/AzFRS and cognate tRNA/GFP150TAG reporter plasmid (1+4 ratio) and cultured in the absence (–ncAA) or presence of 0.25 mM of the indicated ncAA for 24 h. SPAAC labeling with 1 µM DBCO-TAMRA in cell lysate. Lysate aliquots were separated by SDS-PAGE and imaged for in-gel fluorescence at 520 nm. Immunostaining for GFP and FLAG-tagged aminoacyl-tRNA synthetase after membrane transfer of the same gel.

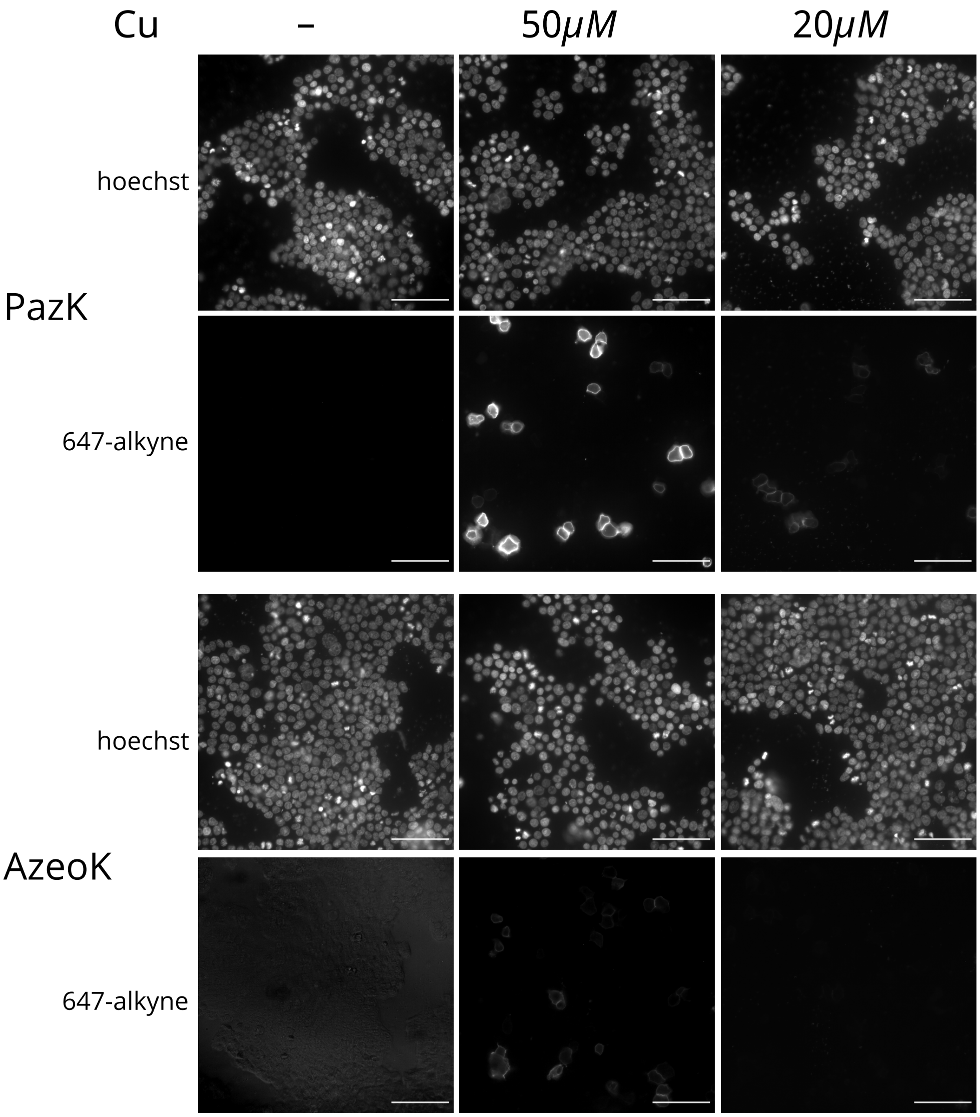

Supplementary Figure 4 - Incorporation of PazK allows CuAAC at reduced copper concentrations

Separate full frame images in grayscale for both hoechst 33342 and AF647 channels of the images in figure 3B. The white scale bar corresponds to 100 μm.

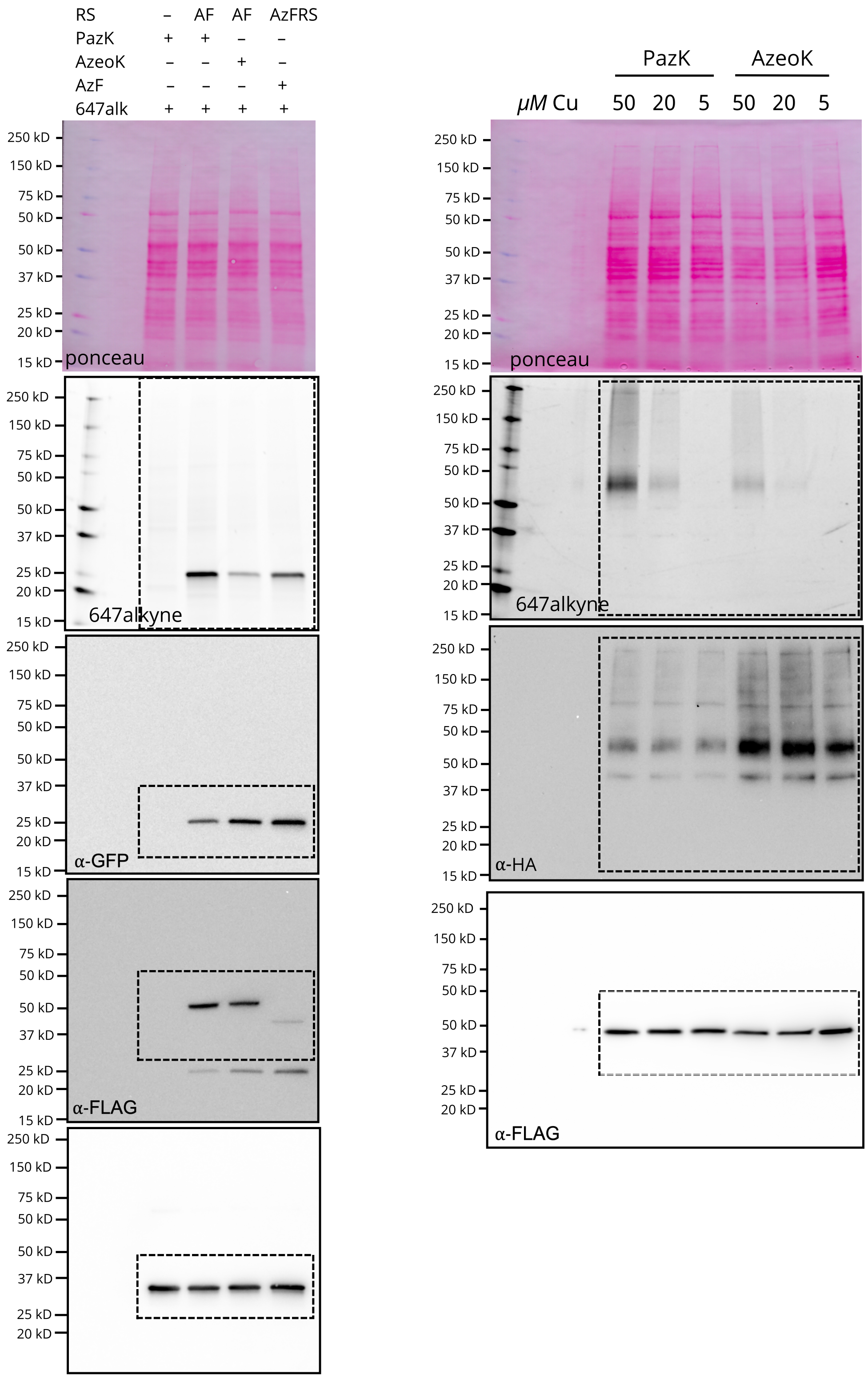

Supplementary Figure 5 – uncropped gel and western blot images to figure 2.

3 Supplementary Chemistry Schemes and Spectra

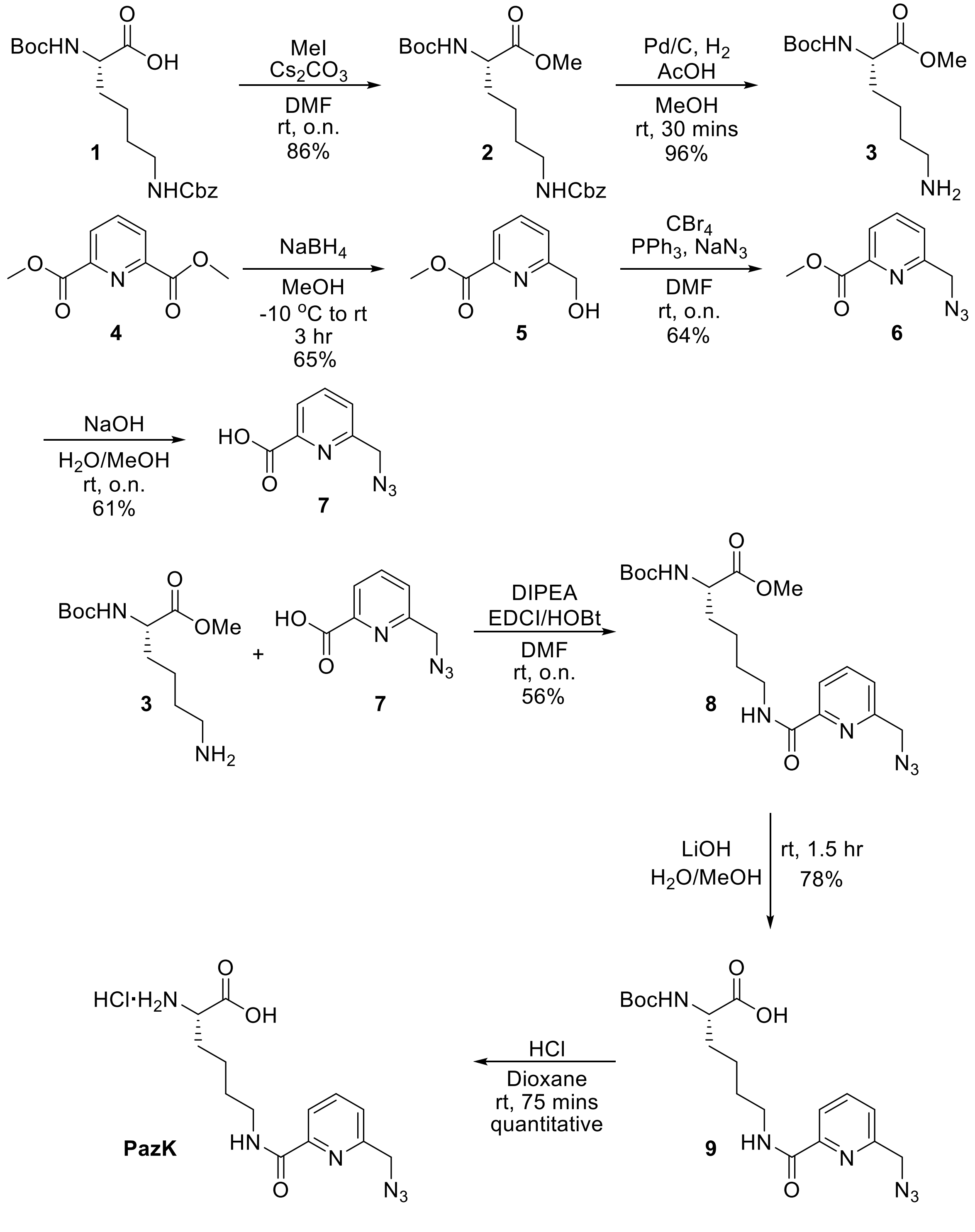

Ester **2**

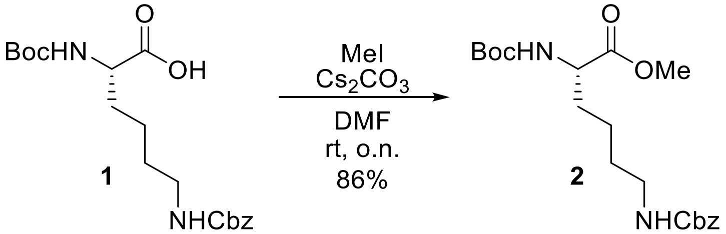

To a solution of **1** (1.00 g, 2.63 mmol) in dry *N*,*N*-dimethylformamide (20 mL) was added cesium carbonate (1.799 g, 5.52 mmol), followed by methyl iodide (0.821 g, 0.360 mL, 5.78 mmol) dropwise at room temperature under a nitrogen atmosphere. The reaction mixture was stirred overnight, before 20 mL of distilled water was added. The resulting solution was extracted with ethyl acetate (x3), before being washed with brine. The resulting organic solution was dried over sodium sulfate, filtered, and reduced *in vacuo*. The crude reaction mixture was purified by silica gel chromatography (1:5 to 2:5 EtOAc: isohexanes) to afford **2** as a clear oil (1.03 g, 86%). Spectroscopic data matched that reported in the literature.[1]

^{1}H NMR (400 MHz, $CDCl_3$) δ: 7.41-7.32 (m, 5H), 5.12 (m, 3H), 4.82 (br s, 1H), 4.31 (app q, 1H, *J* = 5.1 Hz), 3.75 (s, 3H), 3.22 (app q, 2H, *J* = 6.5 Hz), 1.87-1.79 (m, 1H), 1.71-1.52 (m, 3H), 1.46 (s, 9H), 1.33 (m, 2H).

Amine **3**

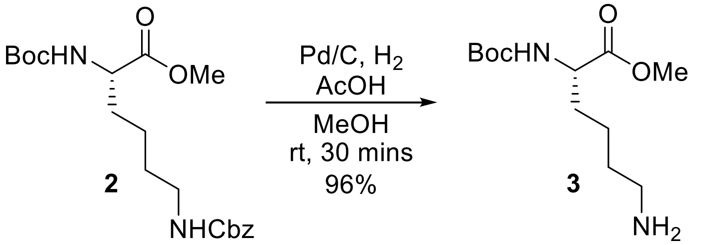

To a solution of **2** (0.459 g, 1.16 mmol) in methanol degassed with nitrogen (pump/purge) at room temperature was added Pd/C (0.046 g, 10% w/w) followed by a drop of acetic acid. The mixture was subjected to a hydrogen atmosphere (pump/purge) and stirred for 30 minutes. At completion, the mixture was degassed with nitrogen (pump/purge), filtered through Celite® (filter cake rinsed with methanol), and concentrated *in vacuo* to afford the title compound as an opaque colorless oil (0.303 g, 96%). Spectroscopic data matched that reported in the literature.[2] No further purification was performed before the material was subsequently reacted.

^{1}H NMR (400 MHz, $CDCl_3$) δ: 5.20 (br s, 1H), 4.28 (app q, 1H, *J* = 5.2 Hz), 3.72 (s, 3H), 2.68 (app q, 2H, *J* = 6.4 Hz), 2.17-1.89 (m, 2H), 1.86-1.72 (m, 2H), 1.69-1.56 (m, 2H), 1.42 (s, 9H).

Alcohol **5**

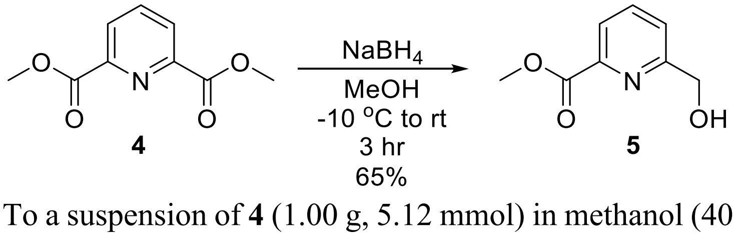

To a suspension of **4** (1.00 g, 5.12 mmol) in methanol (40 mL) cooled to -10 °C in a salted ice bath was added sodium borohydride (0.543 g, 14.3 mmol) in portions over 40 minutes under nitrogen. The mixture was stirred and maintained at -10 °C for two hours, before being diluted with dichloromethane (20 mL) and quenched with saturated aqueous sodium bicarbonate (20 mL). The

organic and aqueous layers were separated (and the current organic layer discarded), and as much methanol as possible was removed *in vacuo* from the aqueous layer. The aqueous layer was then extracted with dichloromethane (x3), before the organic portions were combined and washed with brine, dried over sodium sulfate, filtered, and reduced *in vacuo* to afford **5** as a white powder (856 mg, 65%). Spectroscopic data matched that reported in the literature.[3] No further purification was performed before the material was subsequently reacted.
^{1}H NMR (400 MHz, $CDCl_3$) δ: 8.07 (d, 1H, J = 8.1 Hz), 7.88 (app t, 1H, J = 7.8 Hz), 7.55 (d, 1H, J = 8.2 Hz), 4.99 (m, 2H), 4.03 (s, 3H).

Azide **6**

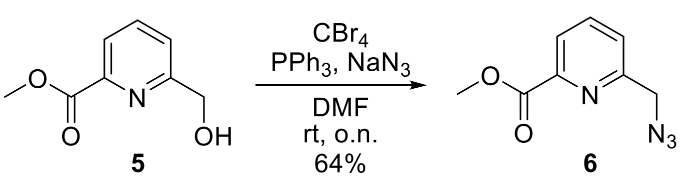

5 **6**

To a solution of **5** (0.201 g, 1.20 mmol) in dry *N*,*N*-dimethylformamide (2 mL) was added triphenylphosphine (0.378 g, 1.44 mmol) followed by carbon tetrabromide (0.479 g, 1.44 mmol) under a nitrogen atmosphere at room temperature. The reaction mixture was stirred for 2 hours, before sodium azide (0.195 g, 3.01 mmol) was added, and the mixture was stirred overnight. At completion, diethyl ether and distilled water were added, before the aqueous layer was extracted with diethyl ether. The organic portions were combined and dried over sodium sulfate, filtered, and reduced in *vacuo*. The crude reaction mixture was purified by silica gel chromatography (1:10 to 2:5 EtOAc: isohexanes) to afford **6** (0.231 g, 64%) as a slightly yellow oil. Spectroscopic data matched that reported in the literature.[4]
^{1}H NMR (400 MHz, $CDCl_3$) δ: 8.12 (d, 1H, J = 7.8 Hz), 7.92 (app t, 1H, J = 7.8 Hz), 7.62 (d, 1H, J = 7.8 Hz), 4.67 (s, 2H), 4.04 (s, 3H).

Acid **7**

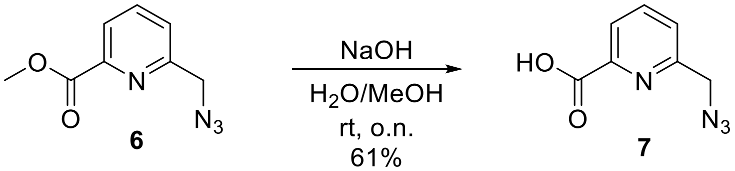

6 **7**

To a solution of **6** (0.149 g, 0.78 mmol) in methanol (4 mL) was added 2M aqueous sodium hydroxide solution (1.5 mL) at room temperature under nitrogen. The mixture was stirred at room temperature overnight, before being acidified to pH 7, and extracted with ethyl acetate (x2). This organic portion was discarded. The aqueous portion was further acidified to pH 3, before being extracted with ethyl acetate (x2), before being washed by saturated aqueous ammonium chloride followed by brine. The organic layer was dried over sodium sulfate, filtered, and concentrated *in vacuo* to afford **7** (0.085 g, 61%) as a white powder. Spectroscopic data matched that reported in the literature.[5]
^{1}H NMR (400 MHz, $CDCl_3$) δ: 8.21 (d, 1H, J = 7.7 Hz), 8.02 (app t, 1H, J = 7.8 Hz), 7.65 (d, 1H, J = 7.7 Hz), 4.58 (s, 2H).

Amide **8**

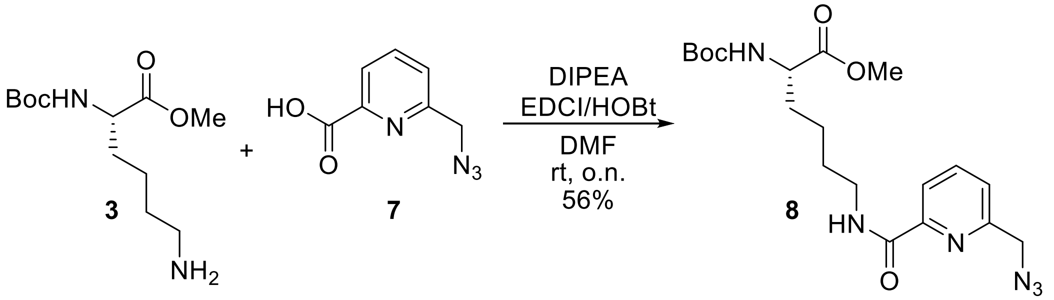

To a solution of **3** (0.124 g, 0.48 mmol) and **7** (0.085 g**,** 0.48 mmol) in *N*,*N*-dimethylformamide (5 mL) was added *N*,*N*-diisopropylethylamine (0.068 g, 0.091 mL, 0.052 mmol), hydroxybenzotriazole (0.080 g, 0.52 mmol), and 1-ethyl-3-(3-dimethylaminopropyl)carbodiimide hydrochloride (0.101 g, 0.525 mmol) at room temperature under nitrogen. The mixture was stirred overnight before being diluted into ethyl acetate (10 mL) and washed with distilled water (x2). The aqueous layers were combined and extracted with ethyl acetate (x2) before the organic portions were combined and washed with brine. The organic layer was dried over sodium sulfate, filtered, and reduced in *vacuo*. The crude material was subjected to silica gel chromatography (1:4 to 1:1 EtOAc: isohexanes) to afford **8** (0.201 g, 56%) as a slightly yellow oil.

^{1}H NMR (400 MHz, $CDCl_3$) δ: 8.16 (d, 1H, *J* = 7.8 Hz), 8.00 (t, 1H, *J* = 5.1 Hz), 7.88 (app t, 1H, *J* = 7.8 Hz), 7.44 (d, 1H, *J* = 7.7 Hz), 5.08 (d, 1H, *J* = 6.8 Hz), 4.47 (s, 2H), 4.30-4.27 (m, 1H), 3.73 (s, 3H), 3.48 (app qd, 2H, *J* = 7.1, 2.5 Hz), 1.90-1.80 (m, 1H), 1.74-1.62 (m, 3H), 1.58-1.55 (m, 2H), 1.43 (s, 9H).

^{13}C NMR (100 MHz, $CDCl_3$) δ: 173.3, 163.9, 156.7, 154.6, 149.8, 138.6, 124.3, 121.6, 79.9, 54.6, 53.3, 52.3, 39.0, 32.3, 29.2, 28.3, 22.7.

HRMS-ESI calculated for $C_{19}H_{28}N_6O_5Na^+$ $[M + Na]^+$: 443.2019; found: 443.2004.

Acid **9**

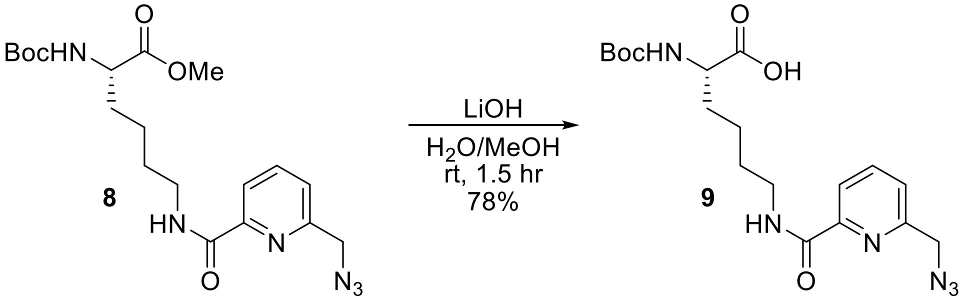

To a solution of **8** (0.016 g, 0.038 mmol) in methanol (0.5 mL) was added 1M aqueous lithium hydroxide (1 mL) at room temperature under nitrogen. The mixture was stirred for 90 minutes, before being acidified to pH 3, and extracted with ethyl acetate (x2). The organic portions were combined and washed with brine, dried over sodium sulfate, filtered, and reduced *in vacuo*. The crude reaction mixture was subjected to silica gel chromatography (1:2:100 AcOH:MeOH:DCM) to afford **9** (0.012 g, 78%) as a clear semi-solid.

^{1}H NMR (400 MHz, $CDCl_3$) δ: 8.16 (d, 1H, *J* = 7.7 Hz), 8.09 (t, 1H, *J* = 5.8 Hz), 7.88 (app t, 1H, *J* = 7.7 Hz), 7.44 (d, 1H, *J* = 7.7 Hz), 5.25 (d, 1H, *J* = 7.6 Hz), 4.47 (s, 2H), 4.33-4.30 (m, 1H), 3.52-3.46 (m, 2H), 1.97-1.88 (m, 1H), 1.82-1.74 (m, 1H), 1.71-1.64 (m, 2H), 1.54-1.48 (m, 2H), 1.43 (s, 9H).

^{13}C NMR (100 MHz, $CDCl_3$) δ: 176.2, 164.8, 156.4, 155.2, 150.2, 139.2, 125.0, 122.3, 80.7, 55.2, 53.8, 39.6, 32.3, 29.7, 28.9, 23.1.

HRMS-ESI calculated for $C_{18}H_{26}N_6O_5Na^+$ $[M + Na]^+$: 429.1862; found: 429.1854.

PazK

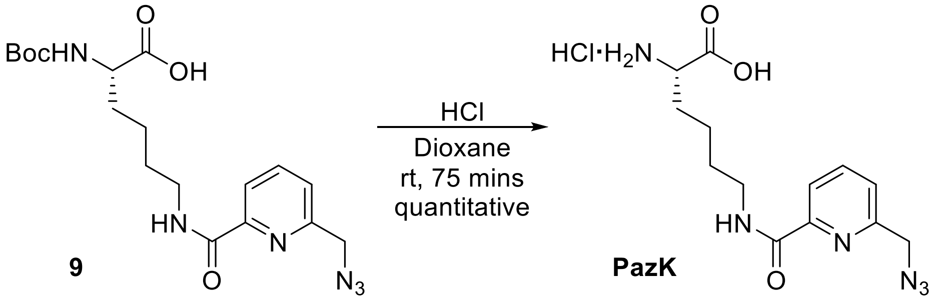

To a flask containing **9** (0.045 g, 0.11 mmol) was added 4 M hydrochloric acid in dioxane (0.5 mL) at room temperature under a nitrogen atmosphere. The mixture was stirred for 75 minutes, before the solvent was removed *in vacuo* to afford **PazK** (32 mg, quantitative) as a white powder.
^{1}H NMR (400 MHz, MeOD) δ: 8.11-8.04 (m, 2H), 7.66 (dd, 1H, J = 7.4, 1.0 Hz), 4.61 (s, 2H), 4.01 (app t, 1H, J = 6.4 Hz), 3.51 (app t, 2H, J = 7.0 Hz), 2.10-1.91 (m, 2H), 1.75 (app p, 2H, J = 7.2 Hz), 1.67-1.49 (m, 2H).
^{13}C NMR (100 MHz, MeOD) δ: 172.4, 166.9, 157.5, 151.3, 140.9, 126.9, 123.0, 56.3, 54.5, 40.7, 31.8, 30.8, 24.1.
HRMS-ESI calculated for $C_{13}H_{18}N_6O_3Na^+$ $[M + Na]^+$: 329.1338; found: 329.1308.

References

1. Wan, W., Huang, Y., Wang, Z., Russell, W. K., Pai, P.-J., Russell, D. H., and Liu, W. R., *Angew. Chem. Int. Ed.,* **2010**, *49* (18), 3211-3214.
2. Schnell, S. D., Hoff, L. V., Panchagnula, A., Wurzenberger, M. H. H., Klapötke, T. M., Sieber, S., Linden, A., and Gademann, K., *Chem. Sci.,* **2020**, *11*, 3042-3047.
3. Zhang, L., Tang, Y., Han, Z., and Ding, K., *Angew. Chem. Int. Ed.,* **2019**, *58* (15), 4973-4977.
4. Vallade, M., Reddy, P. S., Fischer, L., and Huc, Ivan, *Eur. J. Org. Chem.,* **2018**, *40*, 5489-5498.
5. Hanna, J., R., Allan, C., Lawrence, C., Meyer, O., Wilson, N. D., and Hulme, A. N., *Molecules*, **2017**, *22* (5), 802.

^1H NMR (400 MHz, $CDCl_3$)

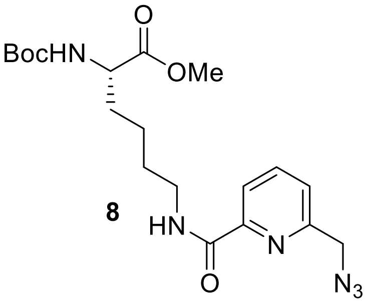

8

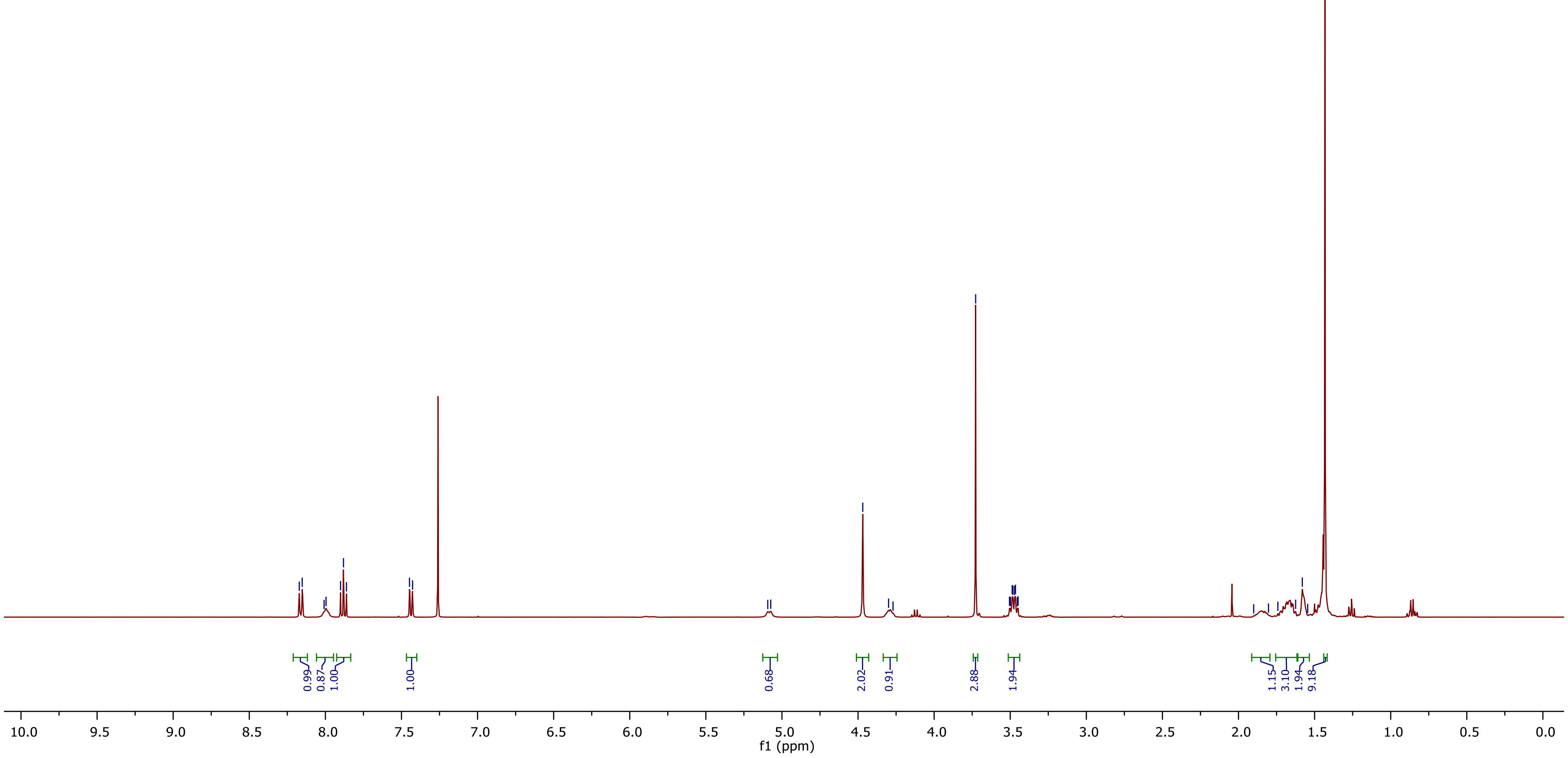

^{13}C NMR (100 MHz, $CDCl_3$)

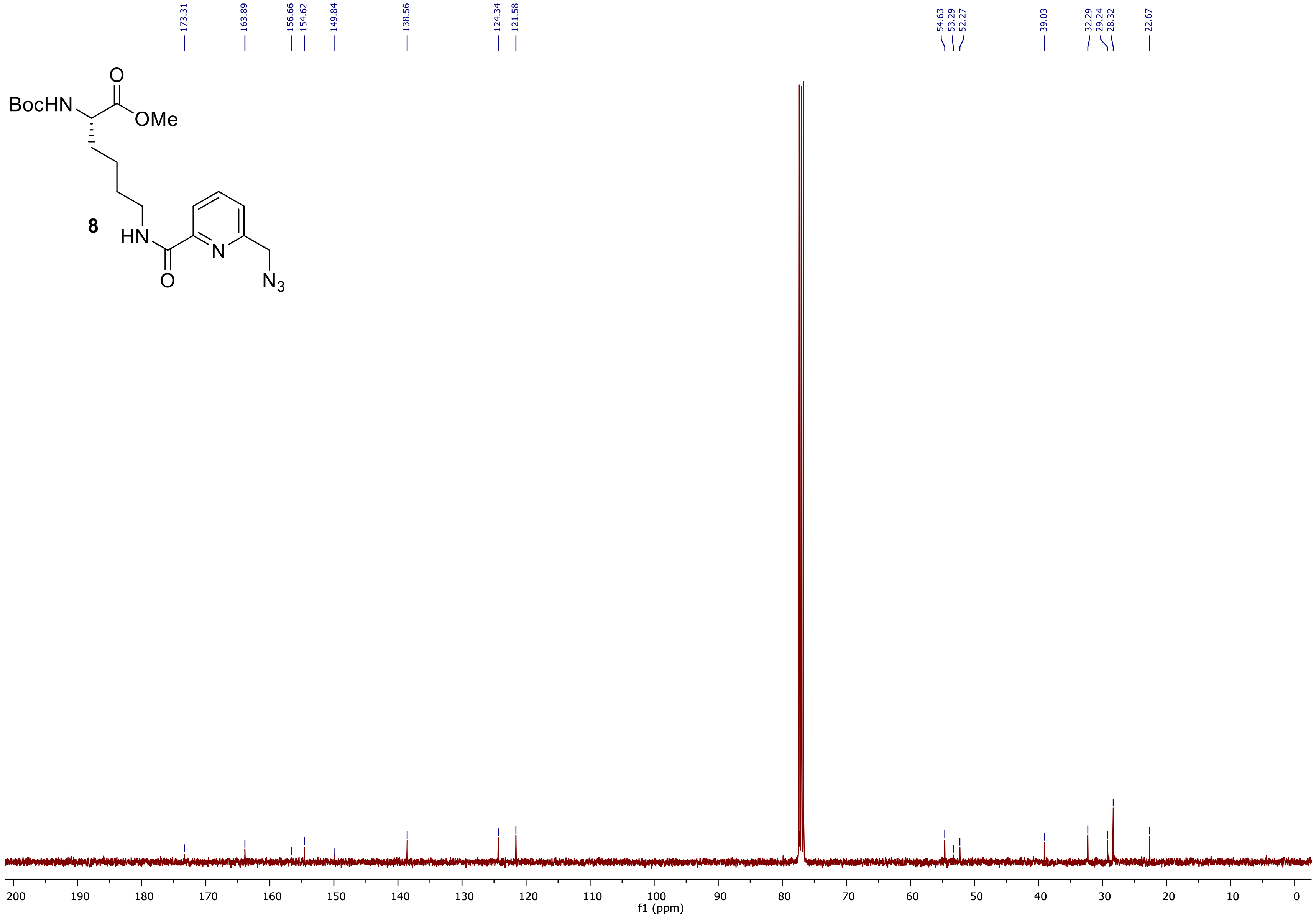

8

^{1}H NMR (400 MHz, CDCl)

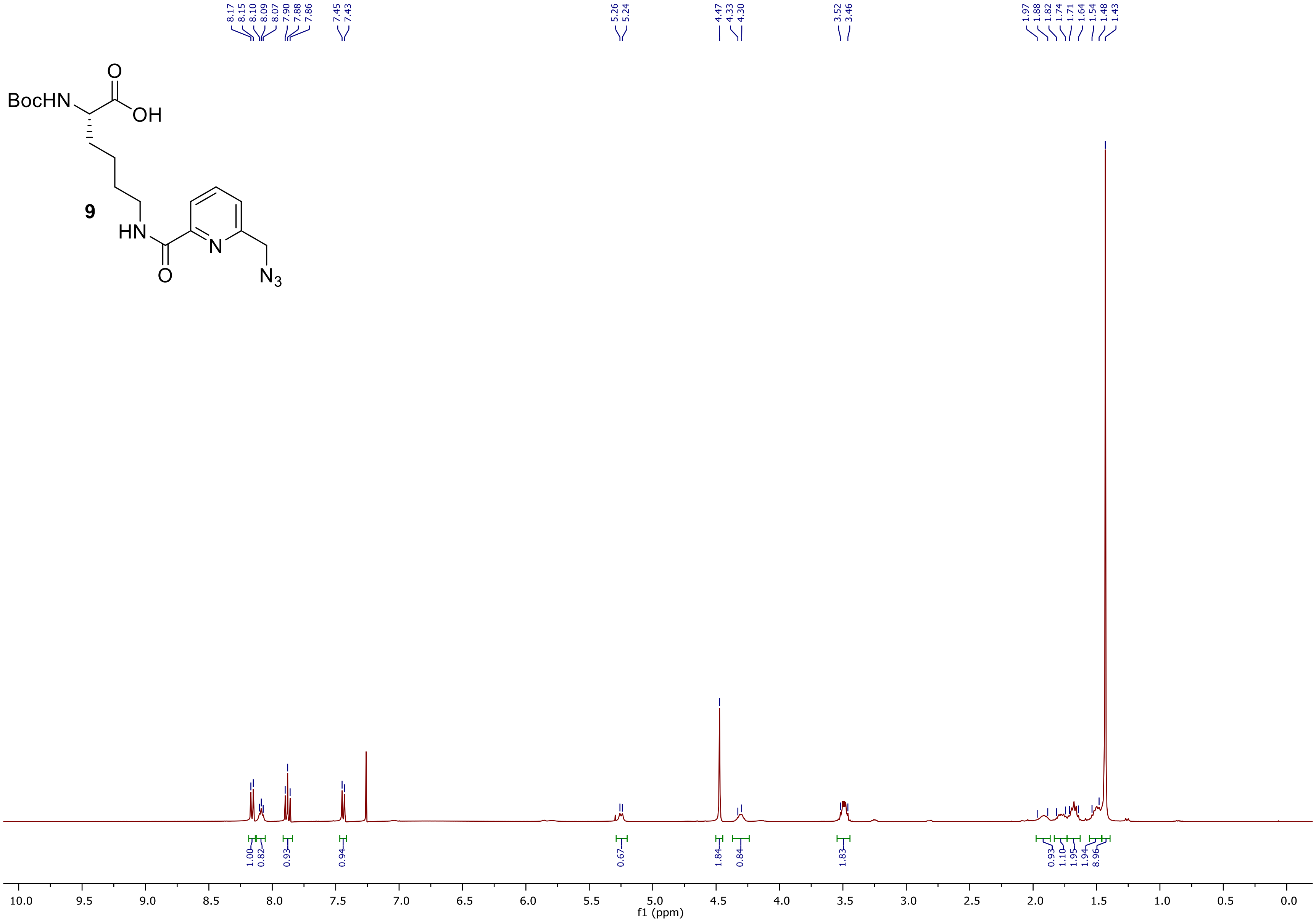

9

^{13}C NMR (100 MHz, $CDCl_3$)

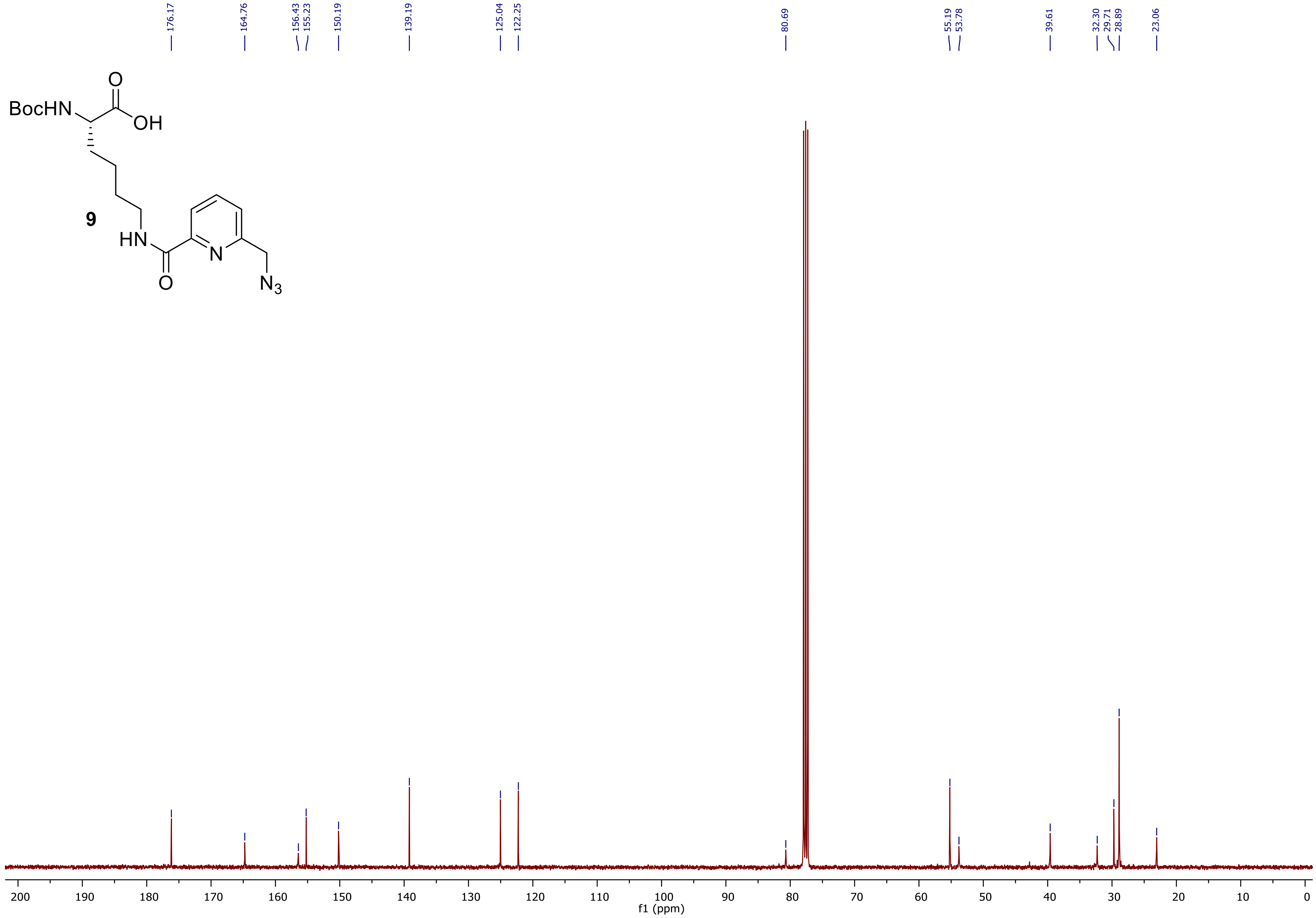

9

^{1}H NMR (400 MHz, MeOD)

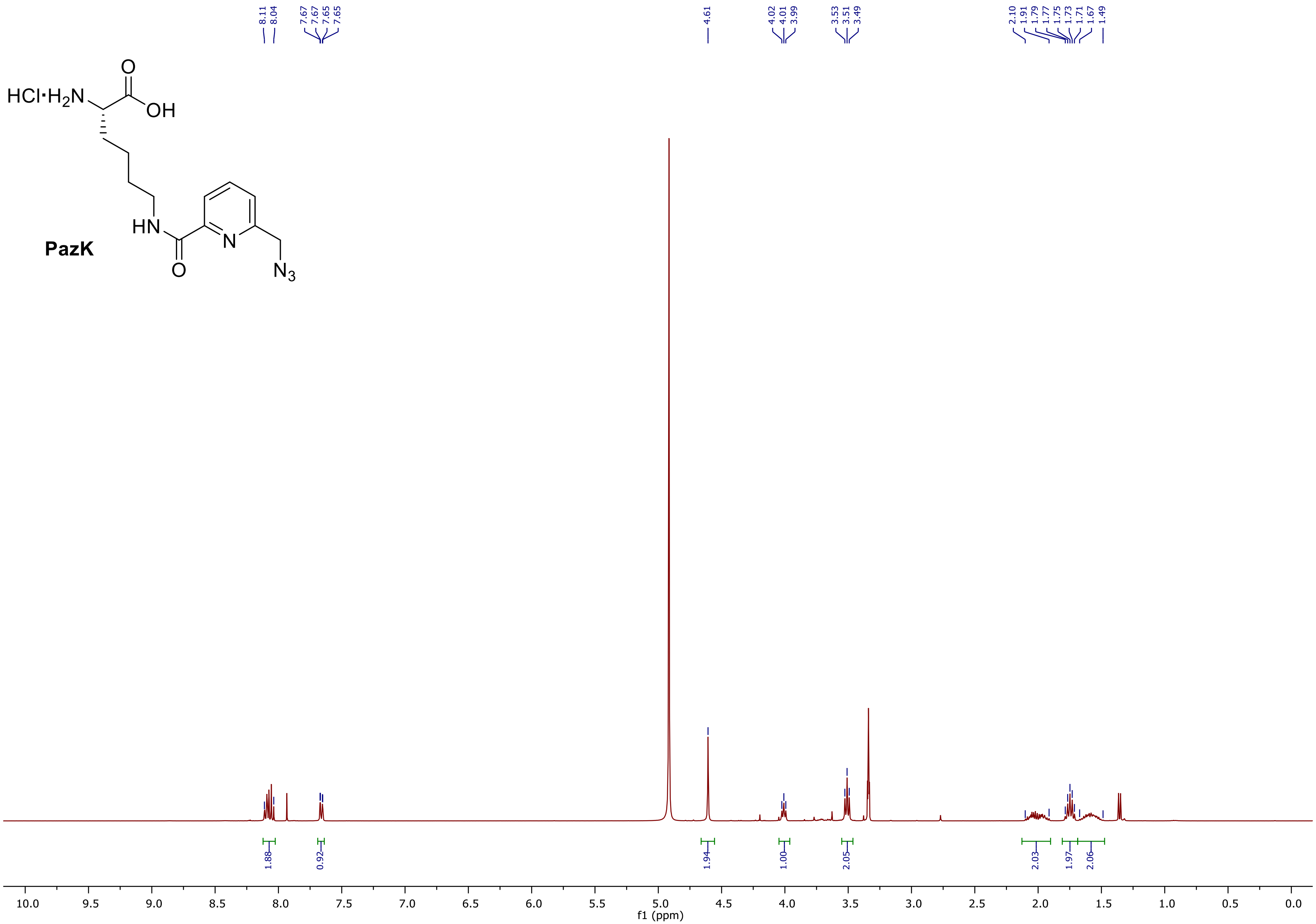

^{13}C NMR (100 MHz, MeOD)

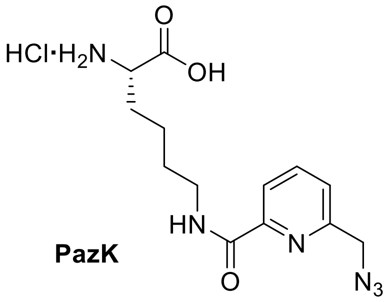

PazK
